# Observations on Intrinsic Capacity and Sarcopenia in Healthcare-Seeking Older Adults

**DOI:** 10.7759/cureus.100595

**Published:** 2026-01-02

**Authors:** Bhrigu Jain, Avinash Chakrawarty, Prasun Chatterjee, Aparajit Ballav Dey, Naval K Vikram, Akash Jaiswal, Maroof Khan

**Affiliations:** 1 Geriatric Medicine, All India Institute of Medical Sciences, New Delhi, New Delhi, IND; 2 Geriatric Medicine, Venu Charitable Society, New Delhi, IND; 3 Medicine, All India Institute of Medical Sciences, New Delhi, New Delhi, IND; 4 Geriatric Medicine, Fortis Memorial Research Institute, Gurugram, IND; 5 Biostatistics, All India Institute of Medical Sciences, New Delhi, New Delhi, IND

**Keywords:** geriatrics, healthcare-seeking older adults, icope, intrinsic capacity, sarcopenia

## Abstract

Introduction

Sarcopenia is a generalized disorder of skeletal muscle associated with adverse outcomes in older adults. With advancing age, the ability to overcome adverse outcomes reduces as measured by reduced intrinsic capacity (IC). Therein lies an underlying pathophysiological relationship between sarcopenia and IC. Our study aimed to explore the association between IC and sarcopenia in healthcare-seeking older adults in India.

Materials and methods

We conducted a cross-sectional study at a tertiary care hospital in New Delhi, India. One hundred and thirty older adults (90 men and 40 women) aged 65 years attended outpatient department services. Sarcopenia was diagnosed as per the Asian Working Group for Sarcopenia (AWGS) 2019 consensus criteria. IC was evaluated as the sum of five domains defined by the World Health Organization (WHO), namely, cognition, sensory (self-reported vision and hearing), locomotion, vitality (nutrition), and psychological. The IC composite score was calculated by summing of scores of all five domains, with a higher score representing lower IC.

Results

The mean age of patients was 70.8±5.8 years. Twenty-six patients (20%) had sarcopenia. Sarcopenic patients were older, less physically active, had a similar incidence of chronic comorbidities, and had significant polypharmacy as compared to non-sarcopenic patients; 73% of total patients had impairment in at least one domain of IC. Cognition, locomotion, sensory, vitality, and psychological domains were impaired in 26%, 48%, 65%, 52% and 44% of total patients, respectively. The median IC composite score was 2 in non-sarcopenic patients, while the sarcopenic patients had a median IC score of 3.5, showing significantly reduced IC. After multivariable analysis, sarcopenic patients had significantly higher impairment in locomotion and vitality domains of IC.

Conclusion

This study showed that lower IC is associated with sarcopenia. Vitality and locomotion domains are strongly associated with sarcopenia. This study is consistent with hypotheses of shared underlying pathophysiology from prior literature, and hence, sarcopenia and IC should be discussed together while diagnosing and managing an older adult in the Integrated Care for Older People (ICOPE) protocol.

## Introduction

Sarcopenia was derived from the Greek phrase “poverty of the flesh” and was first described by Rosenberg in 1989 [1]. Sarcopenia is a progressive and generalized disorder of skeletal muscle associated with advancing age and is associated with increased morbidity and mortality. Worldwide prevalence of sarcopenia in various studies ranges from 10% to 30 % [2]. In India, the prevalence ranges from 15% to 30 % in various community-dwelling and hospital-based studies [3-5]. Sarcopenia is associated with an increased risk of falls, fractures, cognitive impairment, mood disorders, functional impairment, prolonged hospital stay, and higher risk of mortality [6,7]. Hence, sarcopenia poses a significant risk to the healthcare of older adults and is increasingly recognized as a disease warranting urgent screening and management in vulnerable populations. This is particularly relevant given its overlap with frailty, as defined by Fried's criteria, especially through shared physical parameters such as slow walking speed and low grip strength, key indicators of muscle weakness and functional decline that amplify adverse outcomes like falls, disability, and mortality.

The 2015 WHO report on ageing and health [8] defines intrinsic capacity (IC) as “the composite of all the physical and mental capacities that an individual can draw on”. IC comprises five domains of cognition, locomotion, vitality (nutrition), sensory, and psychological capacity. These five domains interact closely with each other and the surroundings of the individual and together form the functional ability of the person. As a combined entity, they reflect the whole health of an older person. In 2017, the WHO published Integrated Care for Older People (ICOPE) guidelines, and an updated version in 2024 [9]. The ICOPE approach embodies the focus on optimizing intrinsic capacity and functional ability as the key to healthy ageing.

Recent evidence suggests an association between sarcopenia and IC [10]. Sarcopenic adults have lower IC, and older adults with impaired domains of IC are more likely to have sarcopenia. The AWGS also recommends that any older adult with functional decline, depressed mood, or malnutrition should be screened for sarcopenia [11]. However, few studies have examined the relationship between IC and its domains with sarcopenia in Indian outpatient settings, leaving a gap in understanding domain-specific associations and their potential role in ICOPE screening. Hence, the present study aimed to explore the relationship between IC, its constituent domains, and sarcopenia and to describe the prevalence and domain-wise distribution of IC impairments and sarcopenia in healthcare-seeking older adults attending a tertiary geriatric outpatient clinic in India.

## Materials and methods

Study design and participants

In this cross-sectional study, healthcare-seeking older adults attending outpatient services of the Department of Geriatric Medicine at All India Institute of Medical Sciences, New Delhi, were recruited via a convenience sampling approach among eligible consecutive attendees. Based on previous estimates of Sarcopenia prevalence in hospital settings [3-5], 130 consenting patients aged 65 years and above (90 men and 40 women) were recruited to yield ~20-40 sarcopenia cases for exploratory analyses from January 2019 to June 2019. Approximately 200 older adults were screened for eligibility, of whom 70 were excluded. Exclusion criteria included decompensated chronic comorbidities (decompensated heart failure or COPD or CKD 4-5, CLD CTP class B or C or as per clinician judgement), advanced dementia, refusal of consent to undergo detailed evaluation, severe osteoarthritis or any metallic implants, coils or pacing devices which impair bioimpedance analysis (BIA). All patients underwent detailed clinical and anthropometric assessment, sarcopenia assessment by BIA, and comprehensive geriatric assessment for IC evaluation.

Covariates

Clinical and socioeconomic data were collected for study participants. Measured covariates included educational status (primary, secondary, or graduate/post-graduate), marital status (married or widowed), smoking history (self-reported current or former smoker vs. never), and physical activity (assessed by a single screening question from the WHO ICOPE toolkit: “Do you perform any regular physical activity or exercise, such as brisk walking, yoga, gardening, cycling, or gym activities, at least 3-4 times per week for 20-30 minutes or more?”; “yes” classified as physically active), and various chronic comorbidities including diabetes mellitus, hypertension, hypothyroidism, and dyslipidemia (all ascertained via self-report confirmed by medical records and current medications). Under geriatric assessment, history of falls (self-reported any unintentional fall to the ground in the last year), urinary incontinence (self-reported any involuntary leakage of urine in the past six months), and polypharmacy (defined as taking ≥5 prescription medications daily, ascertained from medication review) were recorded.

Sarcopenia assessment

Sarcopenia was diagnosed as per the Asian Working Group for Sarcopenia criteria (AWGS) published in 2019 [11]. As per the criteria a diagnosis of sarcopenia was established when the patient had both a low skeletal muscle mass (appendicular skeletal muscle mass index, ASMI, <7.0 kg/m^2^ in men and <5.7 kg/m^2^ in women) and a low muscle strength (hand grip <28 kg in men and <18 kg in women) and /or a low physical performance (gait speed <1.0 m/s). We used a binary categorization (sarcopenia present/absent) for primary analyses due to the study's focus on presence/absence and limited sample size for subgroups; severe sarcopenia as per AWGS (low mass + low strength + low performance) was not separately analyzed in the main models but is explored in a sensitivity analysis. Skeletal muscle mass was assessed using a multi-frequency BIA device BCA-2A, provided by Tsinghua Tongfang Co., Ltd., Beijing, China. It was an eight-electrode direct segmental multi-frequency machine with five testing frequencies (5, 50,100, 250, 500 KHz). Patients were measured in the morning after fasting for at least two hours, with an empty bladder, wearing light clothing, and standing barefoot for at least one minute on the platform with hands and feet in contact with electrodes completing the electrical circuit. A minuscule current of 500μA was passed through this completed circuit, and readings were taken. All metallic ornaments, coins, mobile phones, cashless cards, watches, and glasses were removed before the testing. The device was calibrated daily according to the manufacturer’s instructions using a standard circuit test. ASMI was calculated by dividing the appendicular skeletal muscle mass value by height in m². Hand grip strength was assessed using the Jamar handheld dynamometer. The best of three trials of the dominant hand was taken. Gait speed was measured over the middle 4 meters of the 6-meter walkway after the patients walked at their natural speed. Two consecutive trials were performed, and the faster of the two trials was recorded.

Intrinsic capacity assessment

IC was measured as per WHO guidance as the composite sum of five domains, namely sensory, cognition, locomotion, vitality, and psychological [9]. Cognition was assessed using an ad-hoc Hindi-translated Mini-Cog assessment with a score </=2 indicating cognitive impairment [12]. Locomotion was assessed using the short physical performance battery. A score </=9 indicating impairment [13]. Vitality was assessed using an ad-hoc Hindi-translated DETERMINE checklist with a score >/=3 suggesting risk of malnourishment [14]. The sensory domain was assessed by self-reported vision and hearing ability. Patients were asked if they had experienced any decline in vision and hearing in daily life, even while using their usual glasses and/or hearing aids (if applicable). Those with intact vision and hearing were given 0 score and those with any impairment (in vision or hearing or both) were given a score of 1. Psychological capacity was assessed using an ad-hoc Hindi-translated 5-item Geriatric Depression Scale (GDS-5), with a score of >/=2 indicating impairment [15]. If any domain was impaired, it was given a score of “1”; otherwise, “0”. The composite score of IC was calculated by adding the scores of all five domains. The composite score ranged from 0 to 5, with a higher score indicating lower IC. Impaired IC was defined as having deficits in one or more domains. Furthermore, if these screening tests in any domain were found positive, patients underwent a detailed assessment for further management. Only the Initial screening tests were used to compute IC as per WHO guidance and used in the final analysis. All the tools used for the evaluation of IC and sarcopenia were available in open access, and no special permissions were needed.

Statistical analysis

All statistical analysis was performed using Stata v.16 (StataCorp LLC, College Station, Texas, USA). Continuous variables with normal distribution were expressed as means +/- standard deviation; otherwise, median and interquartile range were used. Categorical variables were expressed as percentages. Baseline characteristics of participants with and without sarcopenia were compared using the t-test for continuous variables with normal distribution or the Mann-Whitney U test for non-normal distribution, while the chi-squared test or Fisher’s exact test (when expected cell count <5) was used for categorical variables. Normality was assessed pragmatically by visual inspection of histograms and Q-Q plots combined with Shapiro-Wilk tests; non-parametric tests were chosen when clear deviation from normality was observed or when sample size in subgroups was limited. Impaired IC domains amongst sarcopenic and non-sarcopenic patients were compared using the chi-squared test, while the composite IC score was compared using the Wilcoxon rank sum test. The IC score and covariates were studied using the Wilcoxon rank sum test or Kruskal-Wallis test for categorical variables or the Spearman correlation coefficient for continuous variables. Multiple logistic regression models were used to examine the independent associations of impaired IC domains and IC composite score with the presence of sarcopenia. Separate models were fitted for each domain plus six covariates (age, sex, physical inactivity, hypertension, diabetes mellitus, dyslipidemia). A single model was fitted with the IC composite score plus the six covariates. Variables were selected a priori based on clinical relevance and univariate associations with p<0.10; no stepwise or automated selection was used. Model fit was assessed using the Hosmer-Lemeshow goodness-of-fit test (p>0.49, indicating good fit) and the area under the ROC curve for indicating good discrimination. There were no missing data for sarcopenia diagnosis, IC composite score, individual IC domains, or any of the covariates included in the regression models; all 130 participants had complete data for these variables. Therefore, no imputation or exclusion was required. All statistical tests were two-sided, and a p-value <0.05 was considered statistically significant. The respective statistical value of the aforementioned tests is mentioned wherever applicable.

## Results

Patient baseline characteristics and sarcopenia

A total of 130 patients were recruited. The mean age of the healthcare-seeking older adults attending the tertiary geriatric outpatient clinic was 70.8±5.8 years. Ninety (69%) patients were male, and 40 (31%) were female. Sarcopenia was found to be present in 26 (20%) patients. The sarcopenia group was significantly older than the non-sarcopenia group, had a higher percentage of widowers and had significantly lower physical activity. Sarcopenic patients had a higher prevalence of polypharmacy, while geriatric syndromes like falls, urinary incontinence and comorbidities like HTN, diabetes mellitus, and dyslipidemia had similar prevalence between the two patient groups (Table 1). All the measures of anthropometry, namely, hand grip strength, gait speed, mid-calf circumference and skeletal muscle mass index were lower among the sarcopenic patients.

**Table 1:** Baseline characteristics comparison among sarcopenic and non-sarcopenic patients HTN: Hypertension; DM: diabetes mellitus; ASMI: appendicular skeletal muscle mass index; SMM: skeletal muscle mass; BMI: body mass index; PBF: percentage body fat; t: t-value; χ²: Chi-square value

Variable	All (130)	Sarcopenia (26)	Non-sarcopenia (104)	Statistic	p-value
	Mean ± SD or N (%)	Mean ± SD or N (%)	Mean ± SD or N (%)		
Age (years)	70.8 ± 5.8	72 ± 6.1	70 ± 5.6	t = 2.03	0.045
Male	90 (69 %)	19 (73%)	71 (68%)	χ² = 0.23	0.630
Female	40 (31%)	7 (27%)	33 (32%)		
Education					
Primary	52 (40%)	14 (54%)	38(36%)	χ² = 2.77	0.250
Secondary	41 (31%)	7 (27%)	34 (33%)		
Graduate	37 (29%)	5 (19%)	32 (31%)		
Marital status					
Married	96 (74%)	14 (54%)	82 (79%)	χ² = 6.84	0.009
Widowed	34 (26%)	12 (46%)	22 (21%)		
Physical activity					
Yes	106 (81%)	14 (54%)	92 (88%)	χ² = 13.87	0.001
Smoking	48 (37%)	11 (42%)	37 (35%)	χ² = 0.44	0.500
HTN	96 (74%)	20 (77%)	76 (73%)	χ² = 0.16	0.900
DM	45 (35%)	8 (31%)	37 (35%)	χ² = 0.20	0.700
Dyslipidemia	20 (15%)	4 (15.4%)	16 (15%)	χ² = 0.00	1.000
Hypothyroidism	21 (16%)	2 (7.7%)	19 (18%)	χ² = 1.77	0.200
Urinary Incontinence	29 (22%)	8 (31%)	21 (20%)	χ² = 1.39	0.240
Falls	47 (36%)	12 46%)	35 (33%)	χ² = 1.46	0.230
Polypharmacy	46 (36%)	14 (54%)	32 (31%)	χ² = 5.20	0.030
Hand grip strength (Kg)	22 ± 7.0	14.6 ± 5.4	23.9 ± 6.3	t = -6.45	0.001
Mid-arm circumference (cm)	24.5 ± 2.4	21.1 ± 2.0	25.3 ± 1.9	t = -8.67	0.001
Mid-calf circumference (cm)	34.3 ± 3.0	30.5 ± 2.1	35.3 ± 2.4	t = -7.81	0.001
Gait speed (m/s)	0.72 ± 0.1	0.65 ± 0.1	0.73 ± 0.1	t = -3.52	0.001
ASMI (Kg/m^2^)	7.6 ± 0.97	6.37 ± 0.79	7.9 ± 0.87	t = -6.77	0.001
SMM (Kg)	29.5 ± 4.9	24.8 ± 4.1	30.6 ± 4.5	t = -6.12	0.001
BMI (Kg/m^2^)	23.7 ± 3.8	19.4 ± 2.6	24.7 ± 3.4	t = -7.03	0.001
PBF (%)	27.9 ± 7.3	23.5 ± 7.3	29.0 ± 6.9	t = -2.86	0.005

Intrinsic capacity and sarcopenia relationship

The IC characteristics (Table 2) showed that the overall prevalence of IC impairment was 73% among all participants. The sarcopenia group had significantly higher prevalence of IC impairment (88%) as compared to the non-sarcopenic group (69%). Similarly, sarcopenic patients had significant impairment in locomotion and vitality domains as compared to the non-sarcopenic group. Furthermore, sarcopenic patients had a higher composite IC score describing overall reduced IC as compared to non-sarcopenic patients.

**Table 2:** Intrinsic capacity composite score and domain-wise impairment among sarcopenic and non-sarcopenic patients IC: Intrinsic capacity; t: t-value; χ²: Chi-square value; U: Mann–Whitney U statistic

Variable	All N (%)	Sarcopenia N (%)	Non sarcopenia N (%)	Statistic	p-value
Impaired IC	95 (73%)	23 (88%)	72 (69%)	χ² = 4.03	0.040
Impaired intrinsic capacity domain (%)					
Cognitive domain	34 (26%)	9 (35%)	25 (24%)	χ² = 1.22	0.270
Sensory domain	85 (65%)	18 (69%)	67 (64%)	χ² = 0.27	0.600
Vitality domain	68 (52%)	24 (92%)	44(42%)	χ² = 21.35	0.001
Locomotor domain	62 (48%)	20 (77%)	42 (40%)	χ² = 10.97	0.001
Psychological domain	57 (44%)	13 (50%)	44 (42%)	χ² = 0.50	0.480
IC composite score {Median (IQR)}		3.5 (3-4)	2 (1-3)	U = 756.0	0.002

On examining the association of the IC composite score and various covariates, we found that IC was significantly lower in female patients, those with advanced age, those with lower educational status, widowers, and those with less physical activity (Tables 3, 4). IC showed a significant correlation with anthropometric parameters with IC decreasing as the anthropometric markers deteriorated. 

**Table 3:** Intrinsic capacity composite score and relationship with various covariates HTN: Hypertension; DM: diabetes mellitus; U: Mann–Whitney U statistic; H: Kruskal–Wallis H statistic

Variable	IC composite score	Statistic	p-value
	Median (IQR)		
Male	2 (1,3)	U = 1428.0	0.040
Female	3 (2,4)		
Education			
Primary	3 (2,4)	H = 13.82	0.001
Secondary	2 (1,3)		
Graduate	2 (1,3)		
Marital status			
Married	2 (1,3)	U = 1270.5	0.008
Widowed	3 (2,4)		
Physical activity			
Yes	2 (1,3)	U = 840.0	0.001
No	4 (3,4)		
Smoking			
Yes	2 (1,3)	U = 1932.0	0.580
No	2 (1,4)		
HTN			
Yes	2 (1,3)	U = 1582.5	0.100
No	3 (2,4)		
DM			
Yes	2 (1,3)	U = 1584.0	0.140
No	2.5 (2,4)		
Dyslipidemia			
Yes	2.5 (1.5, 3.5)	U = 1020.0	0.800
No	2 (1,4)		
Hypothyroidism			
Yes	2 (1,3)	U = 1018.5	0.400
No	2 (1,4)		
Urinary Incontinence			
Yes	3 (2,4)	U = 1357.5	0.200
No	2 (1,3)		
Falls			
Yes	3 (2,4)	U = 1678.0	0.010
No	2 (1,3)		
Polypharmacy			
Yes	3 (1,4)	U = 1836.0	0.200
No	2 (1,3)		

**Table 4:** Intrinsic capacity composite score and relationship with various covariates ASMI: Appendicular skeletal muscle mass index; SMM: skeletal muscle mass; BMI: body mass index; PBF: percentage body fat

Variable	Spearman’s ρ (Coefficient)	p-value
Age	0.18	0.030
Hand grip strength	-0.52	0.001
Mid-arm circumference	-0.39	0.001
Mid-calf circumference	-0.47	0.001
Gait speed	-0.43	0.001
ASMI	-0.51	0.001
SMM	-0.45	0.001
BMI	-0.36	0.001
PBF	-0.03	0.680

While studying the relationship between various domains of IC and various components of sarcopenia (Table 5), we found significantly reduced muscle strength and function as shown by reduced muscle mass, gait speed and grip strength in those with impaired cognition, locomotion and vitality domains. Multivariable logistic regression (Table 6) revealed that, in separate models adjusted for age, sex, physical inactivity, hypertension, diabetes, and dyslipidemia, impaired vitality (adjusted OR 12.4, p=0.002) and locomotion (adjusted OR 4.6, p=0.010) were significantly associated with sarcopenia. Other domains were not significant. A higher IC composite score (indicating lower IC) was associated with sarcopenia (Table 7, adjusted OR per unit 1.72, p=0.030). The multivariable logistic regression models demonstrated good calibration (Hosmer-Lemeshow goodness-of-fit test p>0.49) and discrimination (area under the ROC curve (AUC) 0.82-0.92 for individual domain models in Table 6 and AUC 0.83 for the IC composite model in Table 7). In a sensitivity analysis distinguishing severe sarcopenia (n=12) from non-severe sarcopenia (n=14), median IC scores showed a gradient: 2 (IQR 1-3) for no sarcopenia, 3 (IQR 2-4) for non-severe, and 4 (IQR 3-5) for severe (Kruskal-Wallis p<0.001; Table 8). Multivariable logistic regression for severe sarcopenia (vs. others) showed a stronger association with IC composite score (adjusted OR per unit 2.02, p=0.026; Table 9); (Hosmer-Lemeshow goodness-of-fit test p>0.49) and AUC 0.84).

**Table 5:** Comparison of components of sarcopenia between impaired and intact intrinsic capacity domains ASMI: Appendicular skeletal muscle mass index; t: t-value

Domain	ASMI (Kg/m^2^)	Statistic	p-value	Hand Grip Strength (Kg)	Statistic	P - value	Gait Speed (m/s)	Statistic	p-value
	Mean ± SD			Mean ± SD			Mean ± SD		
Cognition									
Impaired	7 + 0.7	t = -5.41	0.001	18.5 + 6.4	t = -2.86	0.005	0.7 + 0.08	t = -1.71	0.090
Intact	7.8 + 1.0			23.3 + 7.2			0.73+0.1		
Locomotion									
Impaired	7.1 + 0.8	t = -5.67	0.001	17.3 + 6.1	t = -7.82	0.001	0.66+0.1	t = -6.89	0.001
Intact	8 + 1.0			26.3 + 6.5			0.77+0.1		
Vitality									
Impaired	7.2 + 0.9	t = -5.03	0.001	19.7 + 6.8	t = -4.67	0.001	0.7 + 0.09	t = -2.94	0.004
Intact	8 + 0.9			24.6 + 7.0			0.75+0.11		
Sensory									
Impaired	7.6 + 1.0	t = 0.84	0.400	22.4 + 7.1	t = 1.10	0.270	0.72+0.1	t = 0.67	0.500
Intact	7.5 + 0.9			20.8 + 7.3			0.71+0.1		
Psychological									
Impaired	7.4 + 0.9	t = -2.10	0.040	20.5 + 7.0	t = -2.23	0.030	0.7 + 0.1	t = -1.45	0.150
Intact	7.8 + 1.0			23.1 + 7.2			0.73+0.11		

**Table 6:** Multivariable associations of individual IC domain impairments with sarcopenia Adjusted for age, sex, physical activity, hypertension, diabetes mellitus, and dyslipidemia IC: Intrinsic capacity

Impaired domain	Crude OR	95% CI	P - value	Adjusted OR	95 % CI	p-value
Cognition	1.7	0.6-4.2	0.200	0.95	0.3-3.5	0.900
Locomotion	4.9	1.8-12	0.002	4.6	1.3-15.7	0.010
Sensory	1.32	0.5-3.9	0.600	1.6	0.4-5.9	0.500
Vitality	15.1	3.7-60	0.001	12.4	2.5-52	0.002
Psychological	1.36	0.6-3.2	0.500	0.5	0.14-1.8	0.300

**Table 7:** Multivariable association of intrinsic capacity composite score with sarcopenia IC: Intrinsic capacity, adjusted for age, sex, physical activity, hypertension, diabetes mellitus, and dyslipidemia

Variable	Crude OR	95% CI	p-value	Adjusted OR	95 % CI	p-value
IC Composite Score (per unit increase)	1.97	1.35-2.9	0.001	1.72	1.2-2.8	0.030

**Table 8:** Characteristics by sarcopenia severity (sensitivity analysis) χ²: Chi-square value; H: Kruskal–Wallis H statistic; IC: Intrinsic capacity

Variable	No Sarcopenia (n=104)	Non-severe Sarcopenia (n=14)	Severe Sarcopenia (n=12)	Statistic	p-value
Age (years, mean ± SD)	70.3 ± 5.2	71.5 ± 6.0	74.8 ± 6.9	H = 7.15	0.028
IC composite (median [IQR])	2 [1–3]	3 [2–4]	4 [3–5]	H = 16.85	<0.001
Impaired locomotion (%)	40%	71%	100%	χ²= 14.72	0.001
Impaired vitality (%)	48%	86%	100%	χ²= 22.45	<0.001

**Table 9:** Multivariable association of intrinsic capacity composite score with severe sarcopenia (sensitivity analysis) IC: Intrinsic capacity, adjusted for age, sex, physical activity, hypertension, diabetes mellitus, and dyslipidemia

Variable	Crude OR (95% CI)	p-value	Adjusted OR (95% CI)	p-value
IC Composite Score (per unit increase)	2.20 (1.41–3.45)	0.001	2.02 (1.09–3.76)	0.026

## Discussion

Our present exploration is one of the few studies to explore the association between IC and sarcopenia. We found that 73% of the patients had at least one domain impairment in IC. Sarcopenic patients had a significantly higher composite score for IC, denoting poor overall IC. Furthermore, among the domains of IC, locomotion and vitality were found to be strongly associated with sarcopenia after adjusting for covariates. Hence, studying the association between IC and sarcopenia and the shared pathophysiology will help in managing and preventing sarcopenia in clinical practice.

In this study of healthcare-seeking older adults attending the tertiary geriatric outpatient clinic of AIIMS - New Delhi, we found that the prevalence of impaired IC, as measured by impairment of any of the five domains of the construct, was 73%. However, in a recent meta-analysis of nine studies involving participants from various regions all over the world, the pooled prevalence of IC impairment was around 55 % [16]. In a study of hospitalised older adults in China, the prevalence of IC impairment was reported as high as 79 % [17]. Collected data from cohorts of Taiwan Longitudinal Study for Healthy Octogenarians (TLSHO) and the I-Lan Longitudinal Aging Study (ILAS) also showed the prevalence of IC in community-dwelling older adults to be around 79 % [18]. This difference highlights our population as outpatients attending a tertiary geriatric clinic, which sits between community-dwelling and inpatient groups in terms of multimorbidity burden. Variations across studies also reflect measurement differences, such as our use of screening-level binary IC tools versus more stringent continuous or z-score-based IC measures in previous research.

The prevalence of sarcopenia from various studies worldwide ranges from 10% to 40 %. The varied prevalence is in part due to the setting of the study population, muscle mass measurement tools, and the cut-off criterion used. In a meta-analysis of 151 studies, the global prevalence of sarcopenia varied between 10% and 27% [2]. Similarly, in a recent meta-analysis, the global prevalence of sarcopenia ranged from 5 % to 17 %. The prevalence using AWGS criteria was found to be around 15% in the meta-analysis [19]. Indian studies have shown the prevalence to range from 10 % to 36 %, with higher prevalence in inpatient settings and lower prevalence in community-dwelling older adults [3-5]. We found the prevalence of sarcopenia in our study to be 20%. This difference likely reflects both the healthcare-seeking, tertiary-care outpatient nature of our population and the use of the stringent AWGS-2019 diagnostic criteria.

In this study, we found a significant association between the presence of sarcopenia and reduced IC. After adjustment for covariates, individual domains (locomotion and vitality) were also significantly associated with increased risk of sarcopenia. In a cross-sectional study of older adults recruited from the community in Singapore, patients with lower IC had lower appendicular skeletal muscle mass index (ASMI) and higher prevalence of sarcopenia (23.5% vs 5.7%) as compared to those with intact IC [10]. In another cross-sectional study of hospitalised older adults from China, favourable scores in the vitality domain were associated with a greater ASMI, and a higher locomotion score was associated with a greater grip strength. Impairment in IC was associated independently with sarcopenia [17]. In a recent study from Taiwan, the authors found that after adjusting for covariates, participants with sarcopenia had greater impairments in vitality and locomotion domains of IC compared to robust individuals [18]. Moreover, the stronger IC-sarcopenia association in severe cases in our sensitivity analysis suggests a dose-response effect, aligning with AWGS's emphasis on severity in research settings.

All these studies have shown that patients with sarcopenia are more likely to have impairments in locomotion and vitality domains, independent of deficits in other domains of IC. By definition, sarcopenia is diagnosed based on measures of muscle mass and its function; hence, a strong association with the locomotion domain is expected. Strong association with the vitality domain likely arises due to the measurement of vitality using nutritional status and anthropometry, which directly relates to muscle mass and makes the core diagnosis of sarcopenia. Vitality capacity is considered the underlying physiological determinant of IC, and the WHO defines vitality capacity as a physiological state reflected in the level of energy and metabolism, neuromuscular function of the body [20]. Hence, a strong association between sarcopenia and IC may be linked to the vitality domain. The interactions of vitality with other domains of IC and its major role in determining the IC were shown in the INSPIRE ICOPE-Care program, a study of over 14,000 community-dwelling older adults in France. They found a total of 21% adults had impairments in vitality, and the components of vitality (appetite loss and weight loss) were linked to increased risk of impairments in other IC subdomains, especially psychological and locomotion [21]. Furthermore, both possible sarcopenia and sarcopenia were independent predictors for the occurrence of depressive symptoms in a study amongst Chinese older adults [22]. This is in contrast to our findings and likely in part due to fewer older adults in the present study and the different methods of assessment in previous studies and our study. Also, a recent systematic review suggests a potential association between the presence of single or multiple sensory impairments and a greater likelihood of sarcopenia and/or deficits in its associated components, especially for visual and hearing impairment [23]. But we did not observe any association between sensory function and sarcopenia in part due to the sensory assessment being self-reported, rather than objectively measured in the current study. Similarly, assessments carried out on datasets derived through the National Health and Nutrition Examination Survey (NHANES) showed components of sarcopenia (walking speed, ASM, and HGS) were all significantly independently related to cognitive scores [24]. In our study, we also found impaired cognition to be related to low muscle mass and strength, but not amounting to be significantly related to sarcopenia, likely due to the methods of cognitive assessment and baseline variables. Various risk factors for sarcopenia have been mentioned in literature, such as old age, a higher body mass index, or rather high visceral fat, low physical activity, falls, poor nutritional status, smoking, shorter sleep durations, and various comorbidities like diabetes, cardiovascular disease, and neurodegenerative diseases [25]. We also found sarcopenia to be significantly related to increasing age, low physical activity, and polypharmacy amongst the patients. The link between sarcopenia and metabolic diseases is likely due to excessive oxidative stress, insulin resistance, and endothelial dysfunction, causing chronic low-grade inflammation and loss of organ function over an advancing age. While exploring potential pathophysiology for lower IC and sarcopenia, a recent study found an increased level of inflammatory markers such as plasma C-reactive protein, interleukin-6, tumor necrosis factor receptor-1, and growth differentiation factor-15 in individuals with lower IC [26]. Previous studies have shown skeletal muscle wasting is associated with activation of molecular pathways by inflammatory cytokines, inflammatory cell infiltration, leading to muscle protein degradation [27]. Hence, low-grade chronic inflammation associated with lower IC may be linked to muscle wasting and sarcopenia.

Our study and previous studies have shown an association between impaired IC and sarcopenia. Therefore, interventions to preserve or improve IC may potentially be associated with delaying sarcopenia, though this hypothesis requires confirmation in longitudinal and interventional studies. In an RCT from Taiwan, a 12-month multidomain intervention of exercise, cognitive training, and nutritional counselling was associated with significant improvements in overall IC [28]. Similarly, in the TIGER trial, the incorporation of multidomain interventions into community-based care was associated with enhanced IC [29]. The benefit was seen largely in the locomotion and vitality domains. Thus, multidomain interventions targeting nutrition and physical strength are associated with improvements in sarcopenia patients as seen in the network meta-analysis of 59 RCTs, which showed that physical activity, mainly aerobic exercise, and nutrition supplementation were the most effective interventions to improve muscle strength and physical performance [30]. Therefore, sarcopenia prevention and management strategies can offer a multidimensional benefit. It can not only improve energy metabolism but also enhance the vitality domain and potentially other domains of IC.

The major strength of our study lies in the exploration of the relationship between sarcopenia and IC decline, particularly identifying sarcopenia’s link with vitality and locomotion impairment in healthcare-seeking older adults. Use of established AWGS-2019/ICOPE frameworks to explore IC-sarcopenia links in an understudied Indian outpatient setting is another major strength of the study, with domain-specific analyses providing practical insights for ICOPE implementation. The meticulous use of comprehensive geriatric assessment by trained physicians and support staff adds to the quality of data and the observations noted. Nevertheless, the study had some limitations worth mentioning. Firstly, due to the cross-sectional nature of the study and a small sample size, causal associations between IC and sarcopenia cannot be inferred. Also, we did not distinguish primary vs. secondary sarcopenia (e.g., due to comorbidities like diabetes or hypothyroidism), which may confound associations. Secondly, the data on self-reported covariates like chronic diseases, falls, physical activity, and medications may be subject to recall bias. Thirdly, although BIA is a practical, non-invasive method for muscle-mass assessment, it can be influenced by hydration status, recent exercise, or meal timing, potentially leading to overestimation of muscle mass in well-hydrated patients or underestimation in dehydrated ones; this may result in misclassification of sarcopenia, particularly in borderline cases making it less reliable and consistent than dual-emission X-ray absorptiometry. Fourth, widowed status was associated with sarcopenia in univariate analysis, but we did not record the gender of the deceased spouse; thus, we could not examine potential sex-specific effects on IC and sarcopenia risk. Fifth, because the study was conducted in a tertiary-care geriatric outpatient setting, the prevalence of sarcopenia and IC impairments may be higher than in community-dwelling older adults in India, limiting direct generalizability to the broader population. The marked male predominance suggests possible referral or consent biases or sociocultural factors. Selection bias from convenience sampling and unrecorded participation rates may further limit external validity. Sixth, the IC screening tools were translated ad hoc into Hindi without formal local validation or cultural adaptation, which may introduce linguistic or cultural biases and affect the robustness of IC scoring in our Indian outpatient population. Seventh, the multiple unadjusted comparisons in Tables 1-5 carry a non-trivial risk of type I error; thus, bivariate p-values should be viewed as exploratory and hypothesis-generating. Eighth, a low events-per-variable ratio in our logistic regressions risks exaggerated effect sizes. Consequently, the multivariable associations for locomotion and vitality impairments with sarcopenia should be interpreted cautiously. Lastly, no formal power calculation was performed for the primary associations, as this was an exploratory study with convenience sampling. Our study hints towards a common underlying pathobiological cause for sarcopenia and IC. Hence, further studies are needed to understand the impact of sarcopenia and IC on longevity and healthy aging of older adults.

## Conclusions

This cross-sectional study showed that lower IC was associated with a higher prevalence of sarcopenia in healthcare-seeking older adults attending a tertiary geriatric outpatient clinic. Vitality and locomotion domains appeared strongly associated with sarcopenia in adjusted models, though low power limits inference. These associations suggest a possible shared underlying pathophysiology and hence, sarcopenia and IC should be discussed together while diagnosing and managing an older adult in the ICOPE protocol.
